# Short- and Long-term Risks of Highly Active Antiretroviral Treatment with Incident Opportunistic Infections among People Living with HIV/AIDS

**DOI:** 10.1038/s41598-019-39665-6

**Published:** 2019-03-05

**Authors:** Yung-Feng Yen, Marcelo Chen, I.-An Jen, Pei-Hung Chuang, Chun-Yuan Lee, Su-I. Lin, Yi-Ming Arthur Chen

**Affiliations:** 1 Section of Infectious Diseases, Taipei City Hospital, Taipei, Taiwan; 2 Center for Infectious Disease and Cancer Research, Kaohsiung Medical University, Kaohsiung, Taiwan; 3 Department and Institute of Public Health, National Yang-Ming University, Taipei, Taiwan; 4 Department of Health Care Management, National Taipei University of Nursing and Health Sciences, Taipei, Taiwan; 5 Department of Urology, Mackay Memorial Hospital, Taipei, Taiwan; 6 Department of Cosmetic Applications and Management, Mackay Junior College of Medicine, Nursing and Management, Taipei, Taiwan; 7 Taipei Association of Health and Welfare Data Science, Taipei, Taiwan; 8 Division of Infectious Diseases, Department of Internal Medicine, Kaohsiung Medical University Hospital, Kaohsiung Medical University, Kaohsiung, Taiwan; 9 National Mosquito-Borne Diseases Control Research Center, National Health Research Institutes, Miaoli County, Taiwan; 10 Clinical Pharmacogenomics and Pharmacoproteomics Program, School of Pharmacy, Taipei Medical University, Taipei, Taiwan

## Abstract

Highly active antiretroviral therapy (HAART) causes a rapid increase of CD4 + T cells counts during the first 3–6 months of treatment and may enhance the development of opportunistic infections (OIs). However, the short- and long-term effects of HAART exposure on the development of incident OIs has not been extensively studied. This nationwide longitudinal study followed up a total of 26,258 people living with HIV/AIDS (PLWHA) to ascertain the short- and long-term effects of HAART on incident OIs. During 150,196 person-years of follow-up, 6,413 (24.4%) PLWHA had new onset of OIs. After adjusting for demographics, comorbidities, and AIDS status, PLWHA who received HAART were more likely to develop OIs than those who did not receive HAART. Considering the short- and long-term effects of HAART on the development of OIs, HAART was found to be a risk factor for developing OIs during the first 90 days of treatment, but a protective factor against OIs after 180 days of HAART use. The risk for the development of active OIs significantly decreased as the duration of HAART increased (*P* < *0*.*001*). Our study suggests that HAART is a risk factor for developing OIs in the short term, but is a protective factor in the long term.

## Introduction

Acquired immunodeficiency syndrome (AIDS) is a deadly infectious disease caused by the human immunodeficiency virus (HIV). By December 2016, 36.7 million people in the world were living with HIV/AIDS, and more than 35 million having died of the disease^1^.

HIV infection causes the depletion of CD4+ T cells and may increase the risk for opportunistic infections (OIs). OIs have been reported as the major driver of HIV-associated morbidity and mortality, even in the era of highly active antiretroviral therapy (HAART)^2,3^. Since early diagnosis and treatment of OIs could significantly reduce the associated mortality^4^, it is imperative to identify populations at high risk for OIs among PLWHA.

HAART significantly improves survival in PLWHA. Antiretroviral drugs cause a swift increase of CD4+ T lymphocytes during the early phase of treatment. Since immunopathologic host responses to microorganism are central to the clinical presentation of infectious disease, several observational studies found that OIs in PLWHA could develop shortly after HAART initiation^5,6^. However, few longitudinal studies investigated the short- and long-term effects of HAART on incident OIs, and their results were inconsistent. Two studies found that HAART could significantly increase the risk for incident candidiasis or *Mycobacterium avium* complex infection during the first 60 and 90 days of HAART use, respectively^5,7^; however, a US-based study showed that HAART was not significantly associated with a risk for incident tuberculosis (TB) during the first 180 days of HAART^8^. Furthermore, two previous studies revealed that PLWHA who received HAART for more than 90 or 180 days were at lower risk for TB^7,8^, *Mycobacterium avium* complex infection^7^, cryptococcosis^7^, candidiasis^7^, or cytomegalovirus (CMV) infection^7^. However, another study identified no protective effect against candidiasis development in PLWHA after 60 days of HAART^5^.

Identification of risk factors for the development of OIs in PLWHA may help in their prevention and management. We therefore conducted a nationwide longitudinal study to evaluate the short- and long-term effects of HAART on incident OIs among PLWHA in Taiwan.

## Methods

### Data source

In this retrospective longitudinal study, the Taiwan Centers for Disease Control (CDC) HIV Surveillance Database was accessed to identify all reported cases of HIV infection from 2000 to 2014. These reported HIV cases had either a positive HIV-1 polymerase chain reaction or Western blot. In Taiwan, it is mandatory to report all new HIV-infected cases to the Taiwan CDC within 24 hours of diagnosis, and since 1997, free-of-charge HAART has been offered to all HIV-infected individuals^9^. The Taiwan CDC HIV Surveillance Database can only be accessed at the Collaboration Center of Health Information Application, Department of Health, Taiwan, after obtaining approval from the National Health Research Institutes (KMUHIRB-20140073). Patient identification codes in the Taiwan CDC HIV Surveillance Database are scrambled and de-identified before being accessed by the researchers. This study was approved by the institutional review board of Kaohsiung Medical University. The informed consents for study participants were waived in this report. All methods in this study were performed in accordance with relevant guidelines and regulations.

### Study subjects

HIV-infected individuals (age ≥ 15 years) were identified from Taiwan CDC HIV Surveillance Database from 2000 to 2014^10^. These individuals were followed up until a diagnosis of OI was made, December 31, 2014, or death. Death cases were confirmed by the Taiwan death certificate database.

### Outcome variable

The National Health Insurance Research Database in Taiwan was linked to the Taiwan CDC HIV database. Newly diagnosed OIs included disseminated *Mycobacterium avium* complex infection (International Classification of Diseases, Ninth Revision [ICD-9] code 0312), CMV infection (ICD-9-CM code 078.5), *Pneumocystis jirovecii* pneumonia (ICD-9-CM code 1363), cryptococcal meningitis (ICD-9-CM code 3210), candidiasis (ICD-9-CM code 112), *Penicillium marneffei* infection (ICD-9-CM code 1179), and toxoplasma encephalitis (ICD-9-CM code 130). A person was considered to have a new onset of OIs only if the condition occurred in an inpatient setting or if it was recorded in three or more outpatient visits^11^. New onset of tuberculosis was identified by using the Taiwan CDC tuberculosis surveillance database, as medical professionals need to report new cases of turberculosis to Taiwan CDC within 7 days of diagnosis by law.

### Main explanatory variable

HAART was the the main explanatory variable. PLWHA who received HAART were defined as those receiving anti-retroviral drugs before the onset of new OIs. Because of rapid increase of CD4 + T lymphocytes during the first 3–6 months of HAART initiation^12^, the short-term effects of HAART on the development of OIs was defined as the risk of OIs within 90 and between 90 and 180 days of treatment^8^. The long-term effect of HAART was defined as the risk after 180 days of HAART use^8^.

### Other explanatory variables

Sociodemographic variables analyzed included urbanization (urban or rural) and income level. The average monthly income of the insured individual was categorized as: low (≤19 200 New Taiwan Dollars [NTD]), intermediate (19 201 NTD to <40 000 NTD), or high (≥40 000 NTD). The comorbidities investigated included chronic kidney disease (ICD-9 code 580–587), diabetes (ICD-9 code 250), hypertension (ICD-9 code 401–405), congestive heart failure (ICD-9 code 428.0), cerebral vascular disease (ICD-9 code 430–437), cancer (ICD-9 code 140–208), and chronic obstructive pulmonary disease (ICD-9 codes 491, 492, and 496).

AIDS status was defined as the presence of any of the AIDS-defining conditions or a CD4+ lymphocyte count <200 cells/mm^3^ ^13^.

### Statistical analysis

The incidence density (ID) for each OI was calculated in this cohort of PLWHA. To calculate the ID for each OI, PLWHA were excluded if they had received a diagnostic code for the OI before the study period. Moreover, this study evaluated the ID in PLWHA not receiving HAART, those receiving HAART within 90 days, between 90 and 180 days, and after 180 days of treatment. The follow-up years in different phases of treatment were calculated independently in each OI. The ID of each OI was calculated by dividing the number of observed cases by the total person-years at risk for that OI.

Time-dependent Cox proportional hazards models were used to determine the association between HAART and each OI. In these models, death was considered as a competing risk event^14^, and HAART and AIDS status were regarded as time-dependent variables^15^. The short- and long-term effects of HAART on the development of incident OIs were evaluated. Adjusted HRs (AHRs) with 95% CIs were calculated to indicate the direction and strength of associations.

The robustness of the association between HAART and incident OIs was determined by sensitivity analysis, including only subjects with recorded CD4+ count or viral load at the time of HIV diagnosis. Statistical analysis was performed with SAS 9.4 (SAS Institute, Cary, NC).

## Results

### Participant selection

During the period from January 1, 2000 through December 31, 2014, 26 838 HIV-infected individuals were reported to Taiwan CDC. After excluding those younger than 15 years (n = 72) and those with incomplete data (n = 508), the remaining 26 258 PLWHA were included in the analysis (Fig. S1). The overall mean (SD) age was 32.3 (10.2) years; 93.9% of the subjects were male; and 73.4% of the subjects received HAART (Table 1). Compared with PLWHA who did not receive HAART, PLWHA who received HAART had a significantly higher proportion of AIDS status, diabetes, CKD, HTN, COPD, and cancer.

**Table 1:** Characteristics of PLWHA in Taiwan (2000–2014).

Characteristics	PLWHA receiving HAART (n = 19280)	PLWHA not receiving HAART (n = 6978)	P value
**Age**, **yr**			
Mean ± SD	32.45 (10.31)	31.88 (9.73)	<0.001
15–49	17955 (93.13)	6599 (94.57)	<0.001
≥50	1325 (6.87)	379 (5.43)	
**Sex**			
Female	1124 (5.83)	490 (7.02)	<0.001
Male	18156 (94.17)	6488 (92.98)	
**Injecting drug uses**			
No	15749 (81.69)	3773 (54.07)	<0.001
Yes	3531 (18.31)	3205 (45.93)	
**Income level**			
Low	10473 (54.32)	4693 (67.25)	<0.001
Intermediate	6231 (32.32)	1842 (26.40)	
High	2576 (13.36)	443 (6.35)	
**Urbanization**			
Rural	6009 (31.17)	2695 (38.62)	<0.001
Urban	13271 (68.83)	4283 (61.38)	
**AIDS status**			
No	9034 (46.86)	6179 (88.55)	<0.001
Yes	10246 (53.14)	799 (11.45)	
**Comorbidity**			
Diabetes	1040 (5.39)	292 (4.18)	<0.001
Chronic kidney disease	823 (4.27)	224 (3.21)	<0.001
Congestive heart failure	259 (1.34)	77 (1.10)	0.127
Hypertension	2613 (13.55)	568 (8.14)	<0.001
COPD	1140 (5.91)	206 (2.95)	<0.001
Cancer	3153 (16.35)	753 (10.79)	<0.001
Cerebral vascular disease	503 (2.61)	158 (2.26)	0.115
**Follow-up years**			
Mean ± SD	6.03 (3.86)	4.86 (3.55)	<0.001

^*^Unless stated otherwise. PLWHA = people living with HIV/AIDS; SD = standard deviation; HAART = highly active anti-retroviral therapy; AIDS = acquired immunodeficiency syndrome; COPD = chronic obstructive pulmonary disease.

### Incidence of OIs in PLWHA

During the study follow-up period, 6413 (24.4%) PLWHA developed OIs. Of these OIs, *Pneumocystis jirovecii* pneumonia had the highest ID (n = 2978; ID = 21.63/1000 person-years), followed by candidiasis (n = 2658; ID = 19.80/1000 person-years) and CMV infection (n = 893; ID = 6.09/1000 person-years; Table 2).

**Table 2:** Incidence of opportunistic infections in PLWHA.

	New onset of OIs	Follow-up years	ID^*^
Tuberculosis	676	147111.93	4.60
Disseminated MAC	197	149547.58	1.32
CMV infection	893	146750.87	6.09
Pneumocystis jirovecii pneumonia	2978	137697.38	21.63
Cryptococcal meningitis	264	149091.98	1.77
Candidiasis	2658	134215.09	19.80
Penicillium marneffei infection	223	148732.19	1.50
Toxoplasma encephalitis	101	149755.44	0.67

^*^Events per 1,000 person-years. PLWHA = people living with HIV/AIDS; OIs = opportunistic infections; MAC = Mycobacterium avium complex infection; CMV = cytomegalovirus.

### Association between HAART and incident OIs

After adjusting for demographics, comorbidities, and AIDS status, the time-dependent Cox proportional hazards model showed that PLWHA who received HAART were more likely to develop OIs than those who did not receive HAART, such as tuberculosis (AHR 1.88; 95% CI 1.44–2.44), disseminated *Mycobacterium avium* complex infection (AHR 11.7; 95% CI 5.39–25.5), CMV infection (AHR 7.42; 95% CI 5.65–9.74), *Pneumocystis jirovecii* pneumonia (AHR 3.41; 95% CI 2.94–3.94), cryptococcal meningitis (AHR 5.13; 95% CI 3.26–8.09), candidiasis (AHR 2.14; 95% CI 1.86–2.46), *penicillium marneffei* infection (AHR 2.97; 95% CI 1.79–4.93), and toxoplasma encephalitis (AHR 2.84; 95% CI 1.31–6.13; Table 3).

**Table 3:** Hazard ratios of incident opportunistic infections in PLWHA who received HAART and those who did not receive HAART.

	HAART initiation	New onset of OIs	Follow-up years	ID^a^	Unadjusted HR (95% CI)	Adjusted HR (95% CI)^b^
Tuberculosis	No HAART	240	65775.64	3.65	1	1
	HAART	436	81336.29	5.36	3.37 (2.71–4.18)^***^	1.88 (1.44–2.44)^***^
Disseminated MAC	No HAART	8	66121.28	0.12	1	1
	HAART	189	83426.30	2.27	42.2 (20.5–86.7)^***^	11.7 (5.39–25.5)^***^
CMV infection	No HAART	127	66046.15	1.92	1	1
	HAART	766	80704.71	9.49	23.0 (18.5–28.7)^***^	7.42 (5.65–9.74)^***^
Pneumocystis jirovecii pneumonia	No HAART	875	65951.09	13.3	1	1
	HAART	2103	71746.29	29.3	12.2 (10.8–13.7)^***^	3.41 (2.94–3.94)^***^
Cryptococcal meningitis	No HAART	48	66117.11	0.73	1	1
	HAART	216	82974.87	2.6	19.5 (13.5–28.2)^***^	5.13 (3.26–8.09)^***^
Candidiasis	No HAART	994	64894.10	15.3	1	1
	HAART	1664	69320.98	24	6.09 (5.40–6.86)^***^	2.14 (1.86–2.46)^***^
Penicillium marneffei infection	No HAART	52	66009.16	0.79	1	1
	HAART	171	82723.03	2.07	7.17 (4.84–10.6)^***^	2.97 (1.79–4.93)^***^
Toxoplasma encephalitis	No HAART	20	66121.89	0.3	1	1
	HAART	81	83633.55	0.97	10.7 (5.71–20.2)^***^	2.84 (1.31–6.13)^**^

^**^<0.01; ^***^<0.001. ^a^Events per 1,000 person-years. ^b^Adjusted for demographic data, comorbidities, and AIDS status. HAART = highly active anti-retroviral therapy; PLWHA = people living with HIV/AIDS; OIs = opportunistic infections; AHR = adjusted hazard ratio; CI = confident interval; MAC = Mycobacterium avium complex infection; CMV = cytomegalovirus.

### Short- and long-term effects of HAART on incident OIs

The time-dependent Cox proportional hazards model found that PLWHA who received HAART within the first 90 days of treatment were at higher risk of OIs, including TB (AHR 6.92; 95% CI 5.28–9.08), disseminated *Mycobacterium avium* complex infection (AHR 29.4; 95% CI 14.5–59.7), CMV infection (AHR 13.30; 95% CI 10.30–17.1), *Pneumocystis jirovecii* pneumonia (AHR 7.78; 95% CI 6.88–8.79), cryptococcal meningitis (AHR 8.67; 95% CI 5.64–13.30), candidiasis (AHR 5.59; 95% CI 4.91–6.37), *Penicillium marneffei* infection (AHR 6.86; 95% CI 4.14–11.4), and toxoplasma encephalitis (AHR 6.63; 95% CI 2.81–15.6) (Table 4). Moreover, PLWHA who received HAART between 90 and 180 days of treatment had higher risk of incident disseminated *Mycobacterium avium* complex infection (AHR 10.40; 95% CI 4.58–23.7) and CMV infection (AHR 3.54; 95% CI 2.42–5.18). However, when PLWHA received HAART for more than 180 days, they were at lower risk for OIs, including TB (AHR 0.49; 95% CI 0.37–0.64), disseminated *Mycobacterium avium* complex infection (AHR 2.17; 95% CI 1.03–4.58), CMV infection (AHR 0.69; 95% CI 0.51–0.93), *Pneumocystis jirovecii* pneumonia (AHR 0.26; 95% CI 0.23–0.31), cryptococcal meningitis (AHR 0.52; 95% CI 0.31–0.87), and candidiasis (AHR 0.32; 95% CI 0.28–0.37). The risk for the development of active OIs decreased as the duration of HAART increased (*P* < *0*.*001*).

**Table 4:** Hazard ratios of incident opportunistic infections according to the time elapsed since initiation of HAART in PLWHA.

	Time since HAART initiation	New onset of OIs	Follow-up years	ID^a^	Unadjusted HR (95% CI)	Adjusted HR (95% CI)^b^
Tuberculosis	No HAART	240	65775.64	3.65	1	1
	<90 days	240	4467.27	53.72	12.90 (10.40–16.10)^***^	6.92 (5.28–9.08)^***^
	90–180 days	22	4209.60	5.23	2.94 (1.87–4.63)^***^	1.60 (0.98–2.62)
	>180 days	174	72659.42	2.39	0.92 (0.74–1.14)	0.49 (0.37–0.64)^***^
Disseminated MAC	No HAART	8	66121.28	0.12	1	1
	<90 days	91	4554.33	19.98	88.60 (45.30–173.00)^***^	29.40 (14.50–59.7)^***^
	90–180 days	20	4298.39	4.65	38.90 (17.90–84.30)^***^	10.40 (4.58–23.7)^***^
	>180 days	78	74573.58	1.05	9.85 (4.78–20.30)^***^	2.17 (1.03–4.58)^*^
CMV infection	No HAART	127	66046.15	1.92	1	1
	<90 days	520	4428.12	117.4	38.60 (31.30–47.70)^***^	13.30 (10.30–17.10)^***^
	90–180 days	47	4171.16	11.27	11.80 (8.33–16.70)^***^	3.54 (2.42–5.18)^***^
	>180 days	199	72105.43	2.76	3.01 (2.29–3.94)^***^	0.69 (0.51–0.93)^*^
Pneumocystis jirovecii pneumonia	No HAART	875	65951.09	13.27	1	1
	<90 days	1544	4003.77	385.6	26.1 (23.6–28.8)^***^	7.78 (6.88–8.79)^***^
	90–180 days	49	3777.91	12.97	2.83 (2.12–3.78)^***^	0.76 (0.56–1.03)
	>180 days	510	63964.61	7.97	1.23 (1.08–1.41)^**^	0.26 (0.23–0.31)^***^
meningitis	No HAART	48	66117.11	0.73	1	1
	<90 days	147	4531.52	32.44	30.50 (21.60–43.20)^***^	8.67 (5.64–13.30)^***^
	90–180 days	11	4281.21	2.57	7.95 (4.14–15.30)^***^	1.88 (0.93–3.81)
	>180 days	58	74162.14	0.78	2.92 (1.76–4.83)^***^	0.52 (0.31–0.87)^*^
Candidiasis	No HAART	994	64894.10	15.32	1	1
	<90 days	1121	3928.09	285.4	14.90 (13.40–16.60)^***^	5.59 (4.91–6.37)^***^
	90–180 days	46	3693.14	12.46	2.12 (1.57–2.85)^***^	0.78 (0.57–1.06)
	>180 days	497	61699.75	8.06	1.05 (0.92–1.19)	0.32 (0.28–0.37)^***^
Penicillium marneffei infection	No HAART	52	66009.16	0.79	1	1
	<90 days	80	4530.86	17.66	15.4 (10.3–22.9)^***^	6.86 (4.14–11.4)^***^
	90–180 days	9	4280.86	2.10	4.66 (2.28–9.52)^***^	1.99 (0.89–4.47)
	>180 days	82	73911.32	1.11	2.58 (1.72–3.88)^***^	0.90 (0.55–1.47)
Toxoplasma encephalitis	No HAART	20	66121.89	0.30	1	1
	<90 days	45	4557.73	9.87	23.00 (11.60–45.50)^***^	6.63 (2.81–15.6)^***^
	90–180 days	2	4308.42	0.46	3.90 (0.97–15.80)	1.03 (0.23–4.67)
	>180 days	34	74767.40	0.45	2.40 (1.29–4.46)^**^	0.59 (0.30–1.16)

^**^<0.01; ^***^<0.001. ^a^Events per 1,000 person-years. ^b^Adjusted for demographic data, comorbidities, and AIDS status. HAART = highly active anti-retroviral therapy; PLWHA = people living with HIV/AIDS; OIs = opportunistic infections; AHR = adjusted hazard ratio; CI = confident interval; MAC = Mycobacterium avium complex infection; CMV = cytomegalovirus.

### Sensitivity analysis for the association between HAART and incident OIs

A total of 6413 PLWHA with CD4+ counts or viral load data at the time of HIV diagnosis were included in the sensitivity analysis. After adjusting for demographics, comorbidities, AIDS status, CD4+ count, and viral load, HAART was found to be significantly associated with a higher risk of incident OIs, except toxoplasma encephalitis (Supplementary Table 1). Considering the short- and long-term effects of HAART on the development of OIs, PLWHA who received HAART within the first 90 days of treatment were at higher risk of OIs, including tuberculosis, disseminated *Mycobacterium avium* complex infection, CMV infection, *Pneumocystis jirovecii* pneumonia, cryptococcal meningitis, candidiasis, and *Penicillium marneffei* infection (Supplementary Table 2). Moreover, PLWHA who received HAART between 90 and 180 days of treatment had higher risks of incident tuberculosis, disseminated *Mycobacterium avium* complex infection, and CMV infection. However, when PLWHA received HAART for more than 180 days, they were at a lower risk of *Pneumocystis jirovecii* pneumonia and candidiasis. As the duration of HAART increased, the risk of developing active OIs decreased (*P* < *0*.*001*).

## Discussion

This longitudinal study showed that PLWHA who received HAART were more likely to develop OIs than those who did not. Considering the short- and long-term effects of HAART on the development of OIs, HAART was found to be a risk factor for the development of OIs during the first 90 days of HAART, but a protective factor after 180 days of HAART. The risk of incident OIs significantly decreased with increasing duration of HAART.

Previous studies reported that PLWHA could develop OIs soon after starting HAART^5,6^. However, only a few studies investigated the short- and long-term effects of HAART on the development of OIs and reported inconsistent results. A US-based study showed that HAART was not significantly associated with the development of tuberculosis within the first 180 days of treatment (AHR 0.65; 95% CI 0.28–1.51), but was significantly associated with a lower risk of the development of tuberculosis after 180 days of HAART use (AHR 0.29; 95% CI 0.16–0.53)^8^. A French hospital-based study followed up 1647 HIV-infected patients and found that HAART significantly increased the risk for incident candidiasis within the first 2 months of treatment (AHR 2.6; 95% CI 1.2–5.5) but was not significantly associated with the development of candidiasis after 2 months of HAART use (AHR 0.8; 95% CI 0.3–2.4)^5^. Another HIV-CAUSAL Collaboration cohort study found that HAART was a risk factor for *Mycobacterium avium* complex infection during the first 90 days of treatment, but was a protective factor against tuberculosis, *Mycobacterium avium* complex infection, cryptococcosis, candidiasis, and CMV infection after 90 days of HAART use^7^. The present study showed that PLWHA receiving HAART were more likely to develop OIs than those not receiving HAART. While considering the short term and long term effects of HAART on OIs, HAART was found to be a risk factor for the development of OIs during the first 90 days of treatment, but a protective factor after 180 days of treatment. Since the mortality rate was high in PLWHA with OIs^16^, HIV-infected individuals who receive HAART should be followed up carefully for the development of OIs, particularly during the early phase of HAART use.

Biphasic recovery of CD4+ T lymphocyte counts may explain the short-term risk but long-term protection effects of HAART on the development of OIs. When HIV-infected individuals received HAART, the combination antiretroviral drugs cause two stages of CD4+ T lymphocyte recovery, including the rapid increase in CD4+ T cells (CD4 + 5RO^+^) during the first 3–6 months of HAART treatment and a slow increase in CD4+ T cells (CD4 + 5RA^+^, CD62L^+^) after 6 months of HAART use^12,17^. A prior *in vitro* study demonstrated that the CD4+ T lymphocyte count increased 3.5-fold after 3–6 months of HAART and then slowly increased to 4-fold after 2 years of HAART use^12^. The fast increase in CD4+ T lymphocytes following HAART treatment could result in rapid immune restoration. Because OIs often present with an insidious onset in immunodeficient PLWHA^17^, this fast increase in the number of CD4+ T lymphocytes could speed up the development of active OIs by enhancing the immune response to subclinical OIs^5,18^. The slow increase in CD4+ T cells in PLWHA receiving HAART for more than 6 months could lead to a long-term protection against OIs. Since CD4+ T-cell-mediated immunity plays an essential role in protecting against OIs^19^, and the stabilized T cell immunity could provide long-term protection against OIs development in PLWHA receiving HAART for more than 6 months.

This cohort study has several strengths. First, this nationwide study included all HIV-infected subjects with an HIV diagnosis based on a positive HIV-1 polymerase chain reaction or Western blot, which supported the validity of these findings. Second, because all medical costs of PLWHA are covered under the National Health Insurance Program in Taiwan, this cohort study could trace all HIV-infected individuals with referral bias being minimized. Third, unlike previous studies that did not account for changes in HAART exposure during the study period^8^, in this study, HAART was considered a time-dependent variable in order to determine the exact effect of HAART exposure on the development of active OIs^20^. Finally, because the rate of the competing risk of death was high among HIV-infected individuals in this study (12.4%), this report used competing risk analysis to determine the precise association of HAART with incident OIs^21^.

This study also has some limitations. First, because it is not mandatory to report CD4+ counts and viral loads to the Taiwan CDC when reporting new HIV cases, only 6413 (24.4%) cases had these data at the time of HIV diagnosis. The sensitivity analysis showed that PLWHA who received HAART for <90 days were more likely to develop OIs, but were less likely to develop OIs after 180 days of HAART use, after adjusting for comorbidities, CD4+ counts, and viral loads. As the duration of HAART increased, the risk for the development of active OIs significantly decreased. Second, the diagnosis of OIs in this study may be less accurate than diagnoses made in a prospective clinical setting because it was based on administrative claims data recorded by hospitals or physicians Nonetheless, there is no reason to suspect that the validity of claims data would differ on the basis of the patients’ HIV status. This non-differential misclassification of outcome would cause a bias toward a null association. Finally, the generalizability of our findings to other non-Asian ethnic groups needs further verification because most of our subjects were Taiwanese. Nevertheless, our findings may have important clinical implications for improving medical care in PLWHA.

In summary, this nationwide population-based cohort study showed that PLWHA receiving HAART were more likely to develop OIs than those not receiving HAART. HAART was found to be a risk factor for the development of OIs in the short term (during the first 90 days of treatment), but a protective factor in the long run (after 180 days of treatment). Since OIs remain the major driver of HIV-associated morbidity and mortality, our study suggests that PLWHA who received HAART should be monitored carefully for the development of OIs, particularly during the early phase of HAART.

## Supplementary information

Figure S1. Process of enrollment and follow-up among patients with HIV, 2000-2014. PLWHA = people living with HIV/AIDS; HIV = human immunodeficiency virus; OIs = opportunistic infections.

Supplementary table 1. Hazard ratios of incident OIs in PLWHA who received HAART and those who did not receive HAART#.

Supplementary table 2. Hazard ratios of incident OIs according to the time elapsed since initiation of HAART in PLWHA#.
