# Supplementary material for: Short- and Long-term Risks of Highly Active Antiretroviral Treatment with Incident Opportunistic Infections among People Living with HIV/AIDS
Source: Sci Rep. 2019 Mar 5;9:3476. doi: 10.1038/s41598-019-39665-6 (PMC6400900; doi:10.1038/s41598-019-39665-6)
Supplement: Supplementary file 1 — Figure S1. Process of enrollment and follow-up among patients with HIV, 2000-2014. PLWHA = people living with HIV/AIDS; HIV = human immunodeficiency virus; OIs = opportunistic infections. [file 41598_2019_39665_MOESM1_ESM.docx]

**Short- and Long-term Risks of Highly Active Antiretroviral Treatment with Incident Opportunistic Infections among People Living with HIV/AIDS**

**Yung-Feng Yen,^1,2,3,4,#^ Marcelo Chen,^5,6,#^ I-An Jen,^3^ Pei-Hung Chuang,^7^ Chun-Yuan Lee,^8^ Su-I Lin,^3,9^** **&** **Yi-Ming Arthur Chen,^2,10*^**

^1^Section of Infectious Diseases, Taipei City Hospital, Taipei, Taiwan

^2^Center for Infectious Disease and Cancer Research, Kaohsiung Medical University, Kaohsiung, Taiwan

^3^Department and Institute of Public Health, National Yang-Ming University, Taipei, Taiwan

^4^Department of Health Care Management, National Taipei University of Nursing and Health Sciences, Taipei, Taiwan

^5^Department of Urology, Mackay Memorial Hospital, Taipei, Taiwan

^6^Department of Cosmetic Applications and Management, Mackay Junior College of Medicine, Nursing and Management, Taipei, Taiwan

^7^Taipei Association of Health and Welfare Data Science, Taiwan

^8^Division of Infectious Diseases, Department of Internal Medicine, Kaohsiung Medical University Hospital, Kaohsiung Medical University, Kaohsiung, Taiwan

^9^National Mosquito-Borne Diseases Control Research Center , National Health Research Institutes, Taiwan

^10^Department of Microbiology and Institute of Medical Research, College of Medicine, Kaohsiung Medical University, Kaohsiung, Taiwan

^#^Yung-Feng Yen and Marcelo Chen contributed equally to this manuscript.

^*^Address for Correspondence:

Yi-Ming Arthur Chen, MD, ScD, Center for Infectious Disease and Cancer Research, Kaohsiung Medical University, Kaohsiung 807, Taiwan (e-mail: arthur@kmu.edu.tw).

Running head: antiretroviral therapy and opportunistic infections

Word count: 2995

Abstract: 200

Tables: 4

Supplementary figure: 1

Supplementary tables: 2

References: 20

26,258 PLWHA were included

26,838 PLWHA reported in Taiwan, 2000-2014

Excluded (*n* = 580)

- Age < 15 years (*n* = 72)
- HIV patients with incomplete data (*n* = 508)

Follow-up (2000–2014):

150,196 person-years

6413 (24.4%) PLWHA developed OIs

**Figure S1. Process of enrollment and follow-up among patients with HIV, 2000-2014.** PLWHA = people living with HIV/AIDS; HIV = human immunodeficiency virus; OIs = opportunistic infections.
