# Supplementary material for: Short- and Long-term Risks of Highly Active Antiretroviral Treatment with Incident Opportunistic Infections among People Living with HIV/AIDS
Source: Sci Rep. 2019 Mar 5;9:3476. doi: 10.1038/s41598-019-39665-6 (PMC6400900; doi:10.1038/s41598-019-39665-6)
Supplement: Supplementary file 2 — Supplementary table 1. Hazard ratios of incident OIs in PLWHA who received HAART and those who did not receive HAART#. [file 41598_2019_39665_MOESM2_ESM.docx]

**Short- and Long-term Risks of Highly Active Antiretroviral Treatment with Incident Opportunistic Infections among People Living with HIV/AIDS**

**Yung-Feng Yen,^1,2,3,4,#^ Marcelo Chen,^5,6,#^ I-An Jen,^3^ Pei-Hung Chuang,^7^ Chun-Yuan Lee,^8^ Su-I Lin,^3,9^** **&** **Yi-Ming Arthur Chen,^2,10*^**

^1^Section of Infectious Diseases, Taipei City Hospital, Taipei, Taiwan

^2^Center for Infectious Disease and Cancer Research, Kaohsiung Medical University, Kaohsiung, Taiwan

^3^Department and Institute of Public Health, National Yang-Ming University, Taipei, Taiwan

^4^Department of Health Care Management, National Taipei University of Nursing and Health Sciences, Taipei, Taiwan

^5^Department of Urology, Mackay Memorial Hospital, Taipei, Taiwan

^6^Department of Cosmetic Applications and Management, Mackay Junior College of Medicine, Nursing and Management, Taipei, Taiwan

^7^Taipei Association of Health and Welfare Data Science, Taiwan

^8^Division of Infectious Diseases, Department of Internal Medicine, Kaohsiung Medical University Hospital, Kaohsiung Medical University, Kaohsiung, Taiwan

^9^National Mosquito-Borne Diseases Control Research Center , National Health Research Institutes, Taiwan

^10^Department of Microbiology and Institute of Medical Research, College of Medicine, Kaohsiung Medical University, Kaohsiung, Taiwan

^#^Yung-Feng Yen and Marcelo Chen contributed equally to this manuscript.

^*^Address for Correspondence:

Yi-Ming Arthur Chen, MD, ScD, Center for Infectious Disease and Cancer Research, Kaohsiung Medical University, Kaohsiung 807, Taiwan (e-mail: arthur@kmu.edu.tw).

Running head: antiretroviral therapy and opportunistic infections

Word count: 2995

Abstract: 200

Tables: 4

Supplementary figure: 1

Supplementary tables: 2

References: 20

|  | **HAART initiation** | **New onset of OIs** | **Follow-up years** | **ID^a^** | **Unadjusted HR (95% CI)** | **Adjusted HR (95% CI)^b^** |
| --- | --- | --- | --- | --- | --- | --- |
| Tuberculosis | No HAART | 18 | 3893.75 | 4.62 | 1 | 1 |
|  | HAART | 37 | 6638.24 | 5.57 | 8.84 (4.59-17.0)^***^ | 4.26 (1.90-9.58)^***^ |
| Disseminated MAC | No HAART | 2 | 3911.34 | 0.51 | 1 | 1 |
|  | HAART | 35 | 6735.96 | 5.2 | 59.0 (9.98- 349)^***^ | 23.2 (3.51- 154)^**^ |
| CMV infection | No HAART | 33 | 3903.78 | 8.45 | 1 | 1 |
|  | HAART | 189 | 6419.29 | 29.44 | 33.5 (21.5-52.4)^***^ | 13.8 (8.01-23.8)^***^ |
| Pneumocystis jirovecii pneumonia | No HAART | 201 | 3895.13 | 51.6 | 1 | 1 |
|  | HAART | 447 | 5684.88 | 78.63 | 18.9 (15.1-23.7)^***^ | 7.20 (5.48-9.47)^***^ |
| Cryptococcal meningitis | No HAART | 6 | 3911.03 | 1.53 | 1 | 1 |
|  | HAART | 37 | 6708.35 | 5.52 | 18.8 (8.57-41.3)^***^ | 4.70 (1.79-12.3)^**^ |
| Candidiasis | No HAART | 198 | 3829.61 | 51.7 | 1 | 1 |
|  | HAART | 294 | 5670.69 | 51.85 | 8.20 (6.42-10.5)^***^ | 3.05 (2.27-4.09)^***^ |
| Penicillium marneffei infection | No HAART | 6 | 3901.83 | 1.54 | 1 | 1 |
|  | HAART | 24 | 6729.36 | 3.57 | 19.0 (6.61-54.9)^***^ | 10.8 (3.10-38.0)^***^ |
| Toxoplasma encephalitis | No HAART | 2 | 3911.46 | 0.51 |  |  |
|  | HAART | 8 | 6768.21 | 1.18 | 7.93 (1.22-51.3)^*^ | 1.43 (0.11-18.7) |
| **Supplementary table 1.** Hazard ratios of incident OIs in PLWHA who received HAART and those who did not receive HAART^#. **^<.01; ^***^<.001. ^a^events per 1,000 person-years. ^b^Adjusted for demographic data, comorbidities, AIDS status, CD4 count, and viral load. ^#^A total of 6413 PLWHA with available CD4 counts and viral load data at the time of HIV notification were included in the sensitivity analysis. HAART = highly active anti-retroviral therapy; PLWHA = people living with HIV/AIDS; OIs = opportunistic infections; AHR = adjusted hazard ratio; CI = confident interval; MAC = Mycobacterium avium complex infection; CMV = cytomegalovirus. | | | | | | |
