# Supplementary material for: Short- and Long-term Risks of Highly Active Antiretroviral Treatment with Incident Opportunistic Infections among People Living with HIV/AIDS
Source: Sci Rep. 2019 Mar 5;9:3476. doi: 10.1038/s41598-019-39665-6 (PMC6400900; doi:10.1038/s41598-019-39665-6)
Supplement: Supplementary file 3 — Supplementary table 2. Hazard ratios of incident OIs according to the time elapsed since initiation of HAART in PLWHA#. [file 41598_2019_39665_MOESM3_ESM.docx]

**Short- and Long-term Risks of Highly Active Antiretroviral Treatment with Incident Opportunistic Infections among People Living with HIV/AIDS**

**Yung-Feng Yen,^1,2,3,4,#^ Marcelo Chen,^5,6,#^ I-An Jen,^3^ Pei-Hung Chuang,^7^ Chun-Yuan Lee,^8^ Su-I Lin,^3,9^** **&** **Yi-Ming Arthur Chen,^2,10*^**

^1^Section of Infectious Diseases, Taipei City Hospital, Taipei, Taiwan

^2^Center for Infectious Disease and Cancer Research, Kaohsiung Medical University, Kaohsiung, Taiwan

^3^Department and Institute of Public Health, National Yang-Ming University, Taipei, Taiwan

^4^Department of Health Care Management, National Taipei University of Nursing and Health Sciences, Taipei, Taiwan

^5^Department of Urology, Mackay Memorial Hospital, Taipei, Taiwan

^6^Department of Cosmetic Applications and Management, Mackay Junior College of Medicine, Nursing and Management, Taipei, Taiwan

^7^Taipei Association of Health and Welfare Data Science, Taiwan

^8^Division of Infectious Diseases, Department of Internal Medicine, Kaohsiung Medical University Hospital, Kaohsiung Medical University, Kaohsiung, Taiwan

^9^National Mosquito-Borne Diseases Control Research Center , National Health Research Institutes, Taiwan

^10^Department of Microbiology and Institute of Medical Research, College of Medicine, Kaohsiung Medical University, Kaohsiung, Taiwan

^#^Yung-Feng Yen and Marcelo Chen contributed equally to this manuscript.

^*^Address for Correspondence:

Yi-Ming Arthur Chen, MD, ScD, Center for Infectious Disease and Cancer Research, Kaohsiung Medical University, Kaohsiung 807, Taiwan (e-mail: arthur@kmu.edu.tw).

Running head: antiretroviral therapy and opportunistic infections

Word count: 2995

Abstract: 200

Tables: 4

Supplementary figure: 1

Supplementary tables: 2

References: 20

|  | **Time since HAART initiation** | **New onset of OIs** | **Follow-up years** | **ID^a^** | **Unadjusted HR (95% CI)** | **Adjusted HR (95% CI)^b^** |
| --- | --- | --- | --- | --- | --- | --- |
| Tuberculosis | No HAART | 18 | 3893.75 | 4.62 | 1 | 1 |
|  | <90 days | 32 | 1122.78 | 28.50 | 8.89 (4.57-17.3)^***^ | 4.30 (1.93-9.58)^***^ |
|  | 90-180 days | 2 | 1004.36 | 1.99 | 15.6 (2.43- 101)^**^ | 6.52 (1.16-36.5)^*^ |
|  | >180 days | 3 | 4511.11 | 0.67 | 4.78 (0.38-60.2) | 1.15 (0.09-15.0) |
| Disseminated MAC | No HAART | 2 | 3911.34 | 0.51 | 1 | 1 |
|  | <90 days | 24 | 1134.57 | 21.15 | 60.3 (11.6- 313)^***^ | 24.6 (4.57- 133)^***^ |
|  | 90-180 days | 7 | 1014.86 | 6.90 | 60.9 (5.10- 727)^**^ | 14.9 (1.38- 161)^*^ |
|  | >180 days | 4 | 4586.53 | 0.87 | 26.6 (0.28-2541) | 3.00 (0.03- 286) |
| CMV infection | No HAART | 33 | 3903.78 | 8.45 | 1 | 1 |
|  | <90 days | 163 | 1091.62 | 149.32 | 35.4 (22.9-55.0)^***^ | 14.5 (8.60-24.4)^***^ |
|  | 90-180 days | 12 | 973.29 | 12.33 | 19.7 (7.89-49.0)^***^ | 6.32 (2.37-16.9)^***^ |
|  | >180 days | 14 | 4354.38 | 3.22 | 4.18 (1.39-12.6)^*^ | 0.67 (0.22-2.04) |
| Pneumocystis jirovecii pneumonia | No HAART | 201 | 3895.13 | 51.60 | 1 | 1 |
|  | <90 days | 420 | 977.46 | 429.68 | 19.7 (15.7-24.5)^***^ | 7.54 (5.78-9.83)^***^ |
|  | 90-180 days | 10 | 869.38 | 11.50 | 5.11 (2.23-11.7)^***^ | 1.40 (0.56-3.49) |
|  | >180 days | 17 | 3838.03 | 4.43 | 1.89 (0.89-4.02) | 0.29 (0.13-0.61)^**^ |
| Cryptococcal meningitis | No HAART | 6 | 3911.03 | 1.53 | 1 | 1 |
|  | <90 days | 30 | 1130.41 | 26.54 | 18.9 (8.76-40.9)^***^ | 4.83 (1.91-12.2)^***^ |
|  | 90-180 days | 3 | 1012.46 | 2.96 | 23.3 (2.11- 258)^*^ | 3.95 (0.36-43.1) |
|  | >180 days | 4 | 4565.48 | 0.88 | 10.6 (0.48- 233) | 0.92 (0.05-16.4) |
| Candidiasis | No HAART | 198 | 3829.61 | 51.70 | 1 | 1 |
|  | <90 days | 265 | 980.56 | 270.25 | 8.90 (7.03-11.3)^***^ | 3.38 (2.55-4.47)^***^ |
|  | 90-180 days | 13 | 869.82 | 14.95 | 3.31 (1.69-6.46)^***^ | 1.03 (0.51-2.07) |
|  | >180 days | 16 | 3820.31 | 4.19 | 1.10 (0.50-2.40) | 0.25 (0.11-0.53)^***^ |
| Penicillium marneffei infection | No HAART | 6 | 3901.83 | 1.54 | 1 | 1 |
|  | <90 days | 20 | 1130.43 | 17.69 | 22.3 (8.71-56.9)^***^ | 12.2 (4.06-36.9)^***^ |
|  | 90-180 days | 1 | 1013.46 | 0.99 | 2.08 (0.20-21.2) | 0.83 (0.07-9.65) |
|  | >180 days | 3 | 4585.46 | 0.65 | 2.77 (0.35-21.7) | 0.87 (0.08-9.34) |
| Toxoplasma encephalitis | No HAART | 2 | 3911.46 | 0.51 | 1 | 1 |
|  | <90 days | 7 | 1137.34 | 6.15 | 7.67 (1.13-51.9)^*^ | 1.40 (0.10-18.7) |
|  | 90-180 days | 0 | 1019.52 | 0 | 1.03 (0.48-2.21) | 0.12 (0.02-0.84)^*^ |
|  | >180 days | 1 | 4611.36 | 0.22 | - | - |
| **Supplementary table 2.** Hazard ratios of incident OIs according to the time elapsed since initiation of HAART in PLWHA^#^. ^***^<.001. ^a^events per 1,000 person-years. ^b^Adjusted for demographic data, comorbidities, AIDS status, CD4 count, and viral load. ^#^A total of 6413 PLWHA with available CD4 counts and viral load data at the time of HIV notification were included in the sensitivity analysis. HAART = highly active anti-retroviral therapy; PLWHA = people living with HIV/AIDS; OIs = opportunistic infections; AHR = adjusted hazard ratio; CI = confident interval; MAC = Mycobacterium avium complex infection; CMV = cytomegalovirus. | | | | | | |
